# Editorial: Tick-borne Bunyaviruses: an emerging public health threat

**DOI:** 10.3389/fmicb.2025.1564335

**Published:** 2025-02-13

**Authors:** David W. Hawman, Sofia Appelberg, Georg G. Duscher

**Affiliations:** ^1^HDT Bio, Seattle, WA, United States; ^2^Department of Microbiology, The Public Health Agency of Sweden, Solna, Sweden; ^3^Austrian Agency for Health and Food Safety, Institute for Veterinary Disease Control, Moedling, Austria

**Keywords:** Bunyaviruses, risk factors, coagulation parameters, predictive models, vaccine

Bunyaviruses comprise 496 different species (Chen et al., 2023) and the order *Bunyavirales* was recently promoted to the *Bunyaviricetes* class reflecting the expanding number of Bunyaviruses being discovered (Kuhn et al., 2024). These viruses infect a variety of hosts from plants to insects to rodents to humans. Many Bunyaviruses are transmitted to humans via vectors such as ticks, mosquitoes and sandflies for example. In addition, viruses belonging to the *Bunyaviricetes* class are spreading into new areas due to globalization and climate changes and some are on the World Health Organizations list of prioritized diseases (World Health Organization, 2025). Crimean-Congo hemorrhagic fever virus (CCHFV), mostly transmitted by *Hyalomma sp*. ticks, is reported from Southern and Eastern Europe, Africa, the Middle East and Asia, but are now also found in France and Spain (European Centre for Disease Prevention Control, 2025). The *Hyalomma* has also been found as far north as Sweden (Grandi et al., 2020). Heartland virus (HRTV) is found in the South and East of the USA and is transmitted via *Amblyomma americanum* (Dembek et al., 2024). Severe Fever with thrombocytopenia syndrome (SFTS), caused by Dabie bandavirus (DBV) *aka Huaiyangshan Banyangvirus* can be found in China, South Korea and Japan and is vectored mainly by *Haemaphysalis longicornis*, but also other ticks such as *Amblyomma testudinarium*, Japanese hard ticks and *Rhipicephalus microplus* (Casel et al., 2021). All these viruses share an increase of reported cases in humans most probably due to spreading of the vectors and increased human-tick interactions. Additionally, any attempts to predict the course of the diseases in humans are rather difficult and it seems that the treatment of the disease according to the prognoses reflects a crucial factor at an early stage of the disease.

Since the first description of SFTS virus infection in China in 2009 and the identification of the virus in 2011 (Yu et al., 2011), SFTS have represented an upcoming issue due to the severeness of the diseases and its pandemic potential. Although the virus is currently reported from China, Japan, Korea, Vietnam and Taiwan, the main vector tick *H. longicornis* was recently found to occur in the United States of America. Similar to other vector borne viruses, spreading seems very fast due to increase contact of wildlife and human population. Furthermore, migrating birds might play a key role in the spreading of the vector (Casel et al., 2021).

While ticks are the primary vector, SFTS virus can be transmitted to humans from wild animals and pets such as cats and dogs (Seo et al., 2021). Also human-to-human transmission have been reported with transmission via bodily fluids (Kim et al., 2015). After transmission, the incubation time for SFTS is approximately 1–2 weeks. Even though some patients are asymptomatic, the main clinical symptoms are fever, gastrointestinal problems, thrombocytopenia, and leukopenia (Seo et al., 2021). Acute kidney injury and elevated liver enzymes (alanine aminotransferase, aspartate aminotransferase, and alkaline phosphatase) are additional symptoms. However, SFTS can be hard to diagnose since the symptoms are similar to other infectious diseases. Reported mortality rate for SFTS differ between 6.3%−30% (National Institute of Infectious Diseases, China)^1^ and death is primarily due to multiple organ failure. Currently there are a no effective treatment or vaccine for SFTS infection.

With a high mortality rate, global spreading of the vector as well as the lack of a vaccine and scarce knowledge on the biomarkers that goes along with this SFTS, the difficulties with this disease mirrors those of other Bunyaviruses. Thus, research regarding SFTS virus is not only important to improve the knowledge of this specific virus, but can potentially also derive useful information of other Bunyaviruses.

Six showcases are outlined on this topic in this special edition, which might help to elucidate the knowledge on the other vector-borne Bunyaviruses.

The importance of SFTS can be clearly seen in the bibliometric analysis of Zhang and Zhang, who identified an increased interest in research regarding STFS. They identified a clear upward trend, especially from 2021 and forward, in the number of published papers on this topic from the first description. The reason therefor was supposed to be based on multiple factors such as increases severity of cases, more concern regarding the diseases, more case reports and higher demand for prevention and control measures. This work highlights the current trend in SFTS research and enables scientist to remain focussed on these.

One of the approaches to fight against the disease is to elucidate the biomarkers which help to predict the outcome of the diseases. Therefore Liu et al. analyzed risk factors obtained from 24 studies including 4,793 SFTS patients to establish an early prediction model on the outcome of infection and to increase the chance of survival for the patient. The model was validated by an external cohort. Six indicators were identified as relevant parameters: age, hemorrhagic manifestation, encephalopathy, activated partial thromboplastin time, blood urea nitrogen, and serum creatinine. In this study the literature review is used to obtain and analyse knowledge in regard to potential biomarkers and therefore represents the first step toward early prediction models.

Similar, Xia et al. concentrated on eleven coagulation indicators of 40 SFTS patients and highlighted D-Dimer (D-D), von willebrand factor (VWF), and protein S (PS) as the most important once for prediction of the outcome and the severity of disease. Based on the observations of these parameters early interventions can be applied, which might help to reduce the mortality of patients. The authors deliver important informations for closing the knowledge gap in terms of early recognition and intervention of SFTS in data derived from patients.

Even more, He et al. also investigated potential risk factors associated with SFTS. They applied six machine learning (ML) methods on a set of 483 participant's data to construct a prediction model for diseases prognoses and identification of high-risk patient at an early stage of admission. Six variables, age, days from onset to admission, cerebral infarction, calcium ions, creatinine, and creatinine kinase isoenzyme were found to be linked to high risk for fatal course of SFTS patients. The authors state that the use of machine learning can be effective in early detection of patients in risk of dying from SFTS and thus these patients can be managed intensely early in the diseases. The novelty here is the comparison of the use of machine learning tools to be prepared for future analysis and to increase accuracy.

Another important step is to understand the pathogenesis e.g., in animals to increase the understanding of immunosuppression in individuals due SFTSV infection and to support treatment regimes. In this respect, Sakai et al. investigated the germinal center response and the lymph nodes in cats with fatal SFTS, since the pathology in cats with SFTS is rather similar to humans. Here, the impact of the circulating virus in the lymph nodes was discussed and defined as one potential key factor in the pathology of disease. This might help to elucidate the mechanism behind thrombocytopenia, leukopenia, and multi-organ hemorrhage in humans.

Beside prediction attempts based on biomarkers and the understanding of pathology of the course of infection to implement curative treatment, another important countermeasure is to develop an effective vaccine against SFTS. So, Kim et al. tested recombinant protein vaccine candidates based on the nucleocapsid protein (NP) or surface glycoproteins (Gn and Gc) of SFTS virus in a mouse model. The candidates was assessed either alone or in combination and the results show that the combination of Gc and NP represents the most promising candidate so far. In addition, the study also included a longitudinal study of immune responses induced by the vaccine candidates and what factors that might be necessary for long time protection against SFTS infection.

In summary, this special issue highlights the dynamics and approaches in terms of the recent discovered SFTS virus. Based on bibliometric analysis future trends and directions can be observed (Zhang and Zhang). To increase treatment prognosis the identification of biomarkers for the prediction of the outcome of the diseases is obligatory. While comparing literature (Liu et al.) or patient data (Xia et al.) relevant parameters can be defined. By using machine learning and comparison of these machines, prediction will get more accurate (He et al.). Additionally, the understanding of virus pathology (Sakai et al.) is essential to develop effective treatment. Vaccines are also promising countermeasures, as long as protective candidates are found (Kim et al.).

Although mainly focussed on SFTS, these approaches also might guide while performing research on similar topics such as other Bunyavirus.

## Footnotes

^1^National Institute of Infectious Diseases. *Summary of SFTS Cases Reported in the Surveillance of Infectious Diseases*. Available at: https://www.niid.go.jp/niid/ja/sfts/3143-sfts.html (accessed January 27, 2025).
